# Molecular characteristics and analysis of drug resistance-virulence correlation of carbapenem-resistant *Klebsiella pneumoniae* in northern Henan region

**DOI:** 10.1128/spectrum.03164-25

**Published:** 2026-06-10

**Authors:** Min Li, Guofang Li, Zijiang Jia, Liang Zhao, Yalan Yang, Xing'ao Hu, Fan Yang

**Affiliations:** 1 Xinxiang Key Laboratory of Pathogenic Biology, Department of Pathogenic Biology, School of Basic Medical Sciences, Henan Medical University 91593, Xinxiang, Henan, China; National Institute of Science Education and Research, Jatni, Odisha, India

**Keywords:** carbapenem-resistant *Klebsiella pneumoniae*, drug resistance, hypermucoviscous phenotypes, resistance genes, virulence genes

## Abstract

*Klebsiella pneumoniae*, particularly carbapenem-resistant *Klebsiella pneumoniae* (CRKP), has emerged as a pressing threat to global public health. Its intrinsic multidrug resistance and hypervirulence collectively pose a dual burden to clinical management. This study used the modified carbapenem inactivation method (mCIM) and microbroth dilution method to analyze the 225 clinical isolates’ antimicrobial resistance phenotypes to characterize the clinical epidemiology, antimicrobial resistance spectrum, and virulence-associated phenotypes of CRKP isolates in the northern Henan region, which were primarily obtained from intensive care units (ICUs, 51.11%) and sputum specimens (71.56%), with the majority of affected patients aged 60 years or older (61.78%). They exhibited high resistance to β-lactam antibiotics, including imipenem (100%) and meropenem (99.55%), while remaining susceptible to tigecycline (resistance rate of 0.88%). Meanwhile, *bla_CTX-M_* and *bla_KPC_* were the predominant resistance-associated genes, whereas *rmpA2*, *iucA*, *peg*, and *rmpA* served as key virulence-associated markers for hypervirulent CRKP (hv-CRKP). Notably, hv-CRKP showed a significant negative correlation with resistance gene carriage rates (*P* < 0.05) and a significant positive correlation with the hypermucoviscous phenotype (*P* < 0.01). CRKP strains in the northern Henan region exhibit concurrent high-level drug resistance and hypervirulence, with a prevalence of hypervirulent CRKP (hv-CRKP).

**IMPORTANCE:**

Among the prevalent CRKP strains in Northern Henan, the detection rate of hv-CRKP is relatively high. Moreover, the high-mucus phenotype strains are positively correlated with the virulence gene cluster and negatively correlated with the drug resistance gene load. An analysis framework of “drug resistance gene profile–virulence phenotype–molecular association” is necessary to fill the gap in regional research and to provide key data support for building a three-level drug-resistant bacteria prevention and control system across the “national–regional–hospital” levels.

## INTRODUCTION

*Klebsiella pneumoniae*, as a part of the human microbiota, is also a typical opportunistic pathogen. Under specific predisposing conditions, *K. pneumoniae* can cause hospital-acquired and community-acquired infections, with clinical manifestations including pneumonia, bacteremia, wound infections, and urinary tract infections, among others (1). In antimicrobial treatment paradigms, first-line therapeutic regimens typically include fluoroquinolones, aminoglycosides, and cephalosporins. Carbapenem antibiotics are regarded as the last-resort therapeutic option for managing multidrug-resistant infections owing to their broad-spectrum activity and high efficiency.

The global dissemination of carbapenem-resistant *K. pneumoniae* has emerged as one of the most critical public health challenges. Carbapenem resistance and hypervirulence represent two independent evolutionary trajectories that contribute to the persistent spread of *K. pneumoniae* (2). The dissemination of antimicrobial resistance phenotypes is primarily mediated by mobile genetic elements, such as plasmids and transposons, posing a serious threat to clinical treatment. Carbapenemase genes have attracted significant attention as primary drivers of bacterial resistance. Currently known carbapenemase genes mainly include *bla_KPC_*, *bla_NDM_*, *bla_VIM_*, and *bla_OXA-48_*, which are frequently associated with specific hospital-adapted clones and occasionally exhibit complex co-expression relationships with bacterial lineages and antimicrobial resistance determinants (3, 4). The carbapenemases, encoded by these genes belonging to the β-lactamase family, can catalyze the hydrolysis of broad-spectrum β-lactam antibiotics, including meropenem and imipenem, which is the core mechanism by which bacteria develop resistance to carbapenem drugs (5). Although carbapenems (e.g., meropenem and imipenem) are widely used to treat infections caused by bacteria harboring and expressing AmpC β-lactamases and extended-spectrum β-lactamases (ESBLs) (6), their antimicrobial activity is significantly impaired against carbapenemase-producing strains (7).

The primary mechanisms of carbapenem resistance in CRKP involve the following aspects: (i) acquisition of carbapenemase-encoding resistance genes (e.g., *bla_KPC_* and *bla_NDM_*), (ii) production of extended-spectrum β-lactamases or AmpC enzymes, and (iii) absence or structural alteration of outer membrane porins (8). This will significantly reduce the permeability efficiency of carbapenem antibiotics. Epidemiological studies have shown that the outbreak and prevalence of CRKP in specific clonal strains (e.g., ST258) in Europe and North America are closely associated with the high mortality rates of patients (*P* < 0.01) (9, 10). Numerous studies have demonstrated that the mortality rate among critically ill patients infected with CRKP is much higher than that of cases infected with susceptible strains. Data from the China Bacterial Resistance Monitoring Network (CHINET) in 2024 indicated that *K. pneumoniae* has emerged as the second most prevalent isolated bacterium in clinical settings in China, with the resistance rate to imipenem surging from 3.0% in 2005 to 25.5% in 2024, and the resistance rate to meropenem rising from 2.9% to 26.4% during the same period (11). The ongoing increase in CRKP resistance rates nationwide not only limits therapeutic options but also underscores an urgent need for new infection control paradigms. Notably, *K. pneumoniae* readily forms robust biofilms, which enhance its antibiotic resistance (12). Although prior studies have demonstrated that the biofilm-forming ability of CRKP may be relatively weak (13), infections caused by biofilm-forming strains remain challenging to treat (14), which readily facilitates outbreaks and epidemics of widespread.

In recent years, hypervirulent carbapenem-resistant *K. pneumoniae* (hv-CRKP)—a strain that combines hypervirulence with carbapenem resistance—has emerged as a new focus of research. The mortality rate of hv-CRKP-infected patients is significantly higher than that of patients infected with carbapenem-susceptible strains. When CRKP strains carry hypervirulence-associated genes, the infections are more complex and severe (15). Such strains can rapidly breach the host’s defenses to cause severe infections and exhibit widespread resistance to current first-line antimicrobials, leaving the clinical treatment in a dilemma.

Although CRKP resistance testing has been carried out in China, research on CRKP’s molecular characteristics, resistance spectrum, and virulence gene carriage patterns in Northern Henan Province remains scarce. This study aims to conduct molecular analyses on CRKP strains isolated clinically in Northern Henan, focusing on their characteristics, drug resistance gene combination patterns, and the distribution of hypervirulence-associated genes, to provide a theoretical basis for the precise prevention and control of CRKP infections and the optimization of treatment plans in this region.

## MATERIALS AND METHODS

### Sample collection and identification

This study collected 225 non-redundant CRKP isolates from the clinical laboratory departments of three hospitals in the northern Henan region between January 2018 and January 2022. The study used anonymized bacterial isolates from routine clinical laboratory procedures, with no direct patient identifiers and no additional interventions performed on patients; therefore, the requirement for informed consent was waived in accordance with the Declaration of Helsinki. These strains were part of the routine laboratory procedures in the hospital, derived from different clinical specimens (including sputum, blood, urine, secretions, ascites, drainage fluid, pleural fluid, venous catheters, cerebrospinal fluid, and puncture fluid), which were phenotypically identified using the BD Phoenix M50 Automated Bacterial Identification and Antimicrobial Susceptibility Testing System (BD, USA) and preserved at −80°C. Clinical antimicrobial susceptibility testing used *Escherichia coli* ATCC 25922 as the quality control strain. In the modified carbapenem inactivation method (mCIM), *K. pneumoniae* ATCC BAA-1705H and ATCC BAA-1706 were used as positive and negative control strains, respectively.

### Antibiotic sensitivity testing

In this study, the microbroth dilution method, as recommended by the Clinical and Laboratory Standards Institute (CLSI), was used to determine the minimum inhibitory concentration (MIC). The tested antibiotics included cefuroxime (CXM), cefoxitin (FOX), imipenem (IMP), ceftazidime (CAZ), ceftriaxone (CRO), meropenem (MEM), piperacillin-tazobactam (TZP), cefepime (FEP), cefoperazone-sulbactam (SCF), ciprofloxacin (CIP), levofloxacin (LVX), gentamicin (GEN), amikacin (AMI), trimethoprim-sulfamethoxazole (SMZ), and tigecycline (TGC). The drug susceptibility results were interpreted according to the criteria specified in the CLSI M100-S31 (2021) (16). Priority inclusion criteria of antibiotics are as follows: (i) the β-lactam agents mandated for Enterobacteriaceae susceptibility testing, including cephalosporins, carbapenems, and β-lactam/β-lactamase-inhibitor combinations; (ii) fluoroquinolones, aminoglycosides, sulfonamides, and tigecycline that are frequently prescribed in clinical practice; and (iii) agents targeting two key resistance mechanisms, namely extended-spectrum β-lactamase (ESBL) and carbapenemase (17, 18). The antibiotics tested, along with their MIC breakpoints, are detailed in Table 1. The criterion for multidrug resistance (MDR) was resistance to three or more classes of drugs in the drug susceptibility testing.

**TABLE 1:** The resistance rates of 225 CRKP strains to different antibiotics

Drug name	Resistance rate (%)	Explanation and MIC cutoff point, μg/mL			Code officials
		Sensitive	Mediator	Resistance	
CXM	100	≤8	8	≥32	CLSI, 2021
FOX	100	≤4	–^*a*^	≥16	
IMP	100	≤1	–	≥4	
CAZ	100	≤4	8	≥16	
CRO	99.56	≤1	–	≥4	
MEM	99.55	≤1	2	≥4	
TZP	98.67	≤8/4	–	≥32/4	
FEP	98.22	≤2	4-8	≥16	
SCF	96	≤16	32/8	≥64	
CIP	94.22	≤0.25	0.5	≥1	
LVX	92.44	≤0.5	1	≥2	
GEN	85.78	≤2	4	≥8	
AMI	79.56	≤4	8	≥16	
SMZ	67.11	≤2/38	–	≥4/76	
TGC	0.88	≤2	4	≥8	

^*a*^ “–” indicates no intermediate breakpoint according to CLSI 2021 standards.

### ESBL phenotypic confirmation test

The disk diffusion test (DDST, a type of disk diffusion method) was used to detect ESBL phenotypes. First, cultured strains were picked using a sterile inoculating loop and adjusted to a 0.5 McFarland standard (MCF) bacterial suspension, which was uniformly spread onto the surface of Mueller-Hinton (MH) agar plates. Next, the following antibiotic discs were placed on the surface of the medium: cefotaxime (CTX) at 30 μg, cefotaxime/clavulanic acid (CTX/CLA) at 30 μg/10 μg, ceftazidime (CAZ) at 30 μg, and ceftazidime/clavulanic acid (CAZ/CLA) at 30 μg/10 μg. A minimum distance of ≥24 mm was maintained between each disk to prevent overlap of the inhibition zones. The plates were then incubated at 37°C for 18–24 h, after which the diameter of the inhibition zones was measured. If the inhibition zone diameter for the clavulanic acid-containing combination disc is ≥5 mm larger than that of the corresponding single-agent disc, the isolate is determined to be classified as ESBL-positive; otherwise, ESBL-negative.

### Carbapenemase phenotype test

The modified carbapenem inactivation method (mCIM) was performed as follows: a single colony was picked up with a 1 μL calibrated inoculation loop and transferred into a tube containing 2 mL of tryptic soy broth. The tube was vortexed for approximately 15 s to prepare a bacterial suspension. A 10 μg meropenem disc was immersed in the bacterial suspension along the tube wall. The tube was sealed and incubated at 35°C ± 2°C for 4 h. Simultaneously, *E. coli* ATCC 25922 was cultured to the logarithmic growth phase and adjusted to a 0.5 McFarland standard (MCF) suspension, which was uniformly spread onto the surface of MH agar and allowed to air-dry for 5 min. Finally, the meropenem disc was removed, squeezing out excess fluid gently, and placed at the center of the *E. coli* lawn, followed by incubation at 37°C for 18–24 h.

After incubation, if the inhibition zone diameter was 6–15 mm or 16–18 mm with scattered colonies inside the zone, the isolates were classified as carbapenemase-positive; if the inhibition zone diameter was ≥19 mm, they were deemed carbapenemase-negative; and if the inhibition zone diameter was 16–18 mm, or ≥19 mm but with scattered colonies inside the zone, the results were considered indeterminate.

### Biofilm formation detection

Biofilms are complex structures formed by bacteria attaching to surfaces and are associated with drug resistance and environmental adaptability. The crystal violet assay is a commonly used technique to assess biofilm formation ability, with the procedure as follows: first, test strains were prepared as a bacterial suspension at a concentration of 0.5 MCF, and 10 μL of each bacterial suspension was added to an individual well of a 96-well plate, along with 190 μL of sterile LB broth. Triplicate blank control wells were set up for each strain (with 200 μL LB broth). The 96-well plate was then sealed and incubated at 37°C for 18–24 h. After incubation, the supernatants were discarded, and each well was washed three times with 200 μL of sterile water to remove planktonic (non-adherent bacteria). The adhered biofilms were fixed with 200 μL 99% methanol for 15 min, then stained with 200 μL of 1% crystal violet for 20 min, after which the staining solution and the plate were rinsed three times with sterile water and air-dried at room temperature. Next, 200 μL of 95% ethanol was added to each well, shaken to decolorize to ensure complete dissolution. Finally, a microplate reader was used to measure each well’s absorbance (optical density [OD] value) at 570 nm. The cutoff value was set as the average absorbance of the blank control group (Ac) plus three times the standard deviation (SD) of the blank control group (Ac + 3SD) (19, 20). If OD_570_ >2× cutoff, it is positive; otherwise, it is negative.

### Hypermucoviscous phenotype detection

The hypermucinous phenotype (Hypermucoviscous, HM) refers to the characteristic of bacteria synthesizing excessive mucinous polysaccharides during growth, and its expression is closely associated with bacterial virulence, antimicrobial resistance, and environmental adaptability. The string test can directly measure the mucus secretion capacity of a strain. Test bacteria strains were uniformly streaked onto MacConkey agar plates and incubated at 37°C for 18–24 h. A single colony was picked using a sterile inoculating loop, and the resulting mucus filament was gently stretched vertically upward. If the mucoid string extended more than 5 mm from the colony, it was classified as HM-positive; otherwise, it was designated as non-hypermucoviscous (non-HM).

### Detection of drug resistance genes and virulence genes

Genomic DNA of *K. pneumoniae* strains was extracted from bacterial isolates using the boiling method and then used as a template to amplify drug resistance and virulence genes by Polymerase Chain Reaction (PCR). The drug resistance genes included ESBL genes (*bla_TEM_*, *bla_SHV_*, and *bla_CTX-M_*), carbapenemase genes (*bla_KPC_*, *bla_IMP_*, *bla_VIM_*, *bla_NDM_*, and *bla_OXA-48_*), and virulence-associated genes (*peg-344*, *rmpA*, *rmpA2*, *iroB*, and *iucA*). Generally, strains harboring any four hypervirulence-defining genes (*rmpA*, *rmpA2*, *iucA*, *iroB*, and *peg-344*) are defined as hypervirulence strains (21). The primer sequences of drug resistance and virulence genes are listed in Table 2. The PCR cycling conditions were as follows: initial denaturation at 94°C for 4 min; 32 cycles of denaturation at 94°C for 30 s, annealing at (Tm-3)°C for 30 s, and extension at 72°C for 1 min, followed by a final extension at 72°C for 10 min. Each run included positive controls (*K. pneumoniae* ATCC BAA-1705 for resistance genes, and NTUH-K2044 for virulence genes), a negative control (*E. coli* ATCC 25922), and a no-template control (sterile water) (22, 23). PCR amplicons were separated by 1% agarose gel electrophoresis and visualized using a gel documentation system.

**TABLE 2:** Primer sequence for antimicrobial resistance genes and virulence genes

Genes	Primer sequence (5′–3′)	Product size (bp)	Ta opt (°C)
*bla_KPC_*	F: CGTCTAGTTCTGCTGTCTTG	798	58
	R: CTTGTCATCCTTGTTAGGCG		
*bla_CTX-M_*	F: ACAGCGATAACGTGGGGTATG	216	56
	R: TCGCCAATGCTTTACCCAG		
*bla_SHV_*	F: ATGCGTTATATTCGCCTGTG	753	58.4
	R: TGCTTTGTTATTCGGGCCAA		
*bla_TEM_*	F: TCGCCGCATACACTATTCTCAGAATGA	445	55.1
	R: ACGCTCACCGGCTCCAGATTTAT		
*bla_NDM_*	F: CCAGCTCGCACCGAATG	564	58.8
	R: AACGCCGCACCAAACG		
*bla_IMP_*	F: CTTGATGAAGGCGTTTATGT	496	50.9
	R: GCCAAGCTTCTATATTTGCGT		
*bla_OXA-48_*	F: ACATAAATCACAGGGCGTAG	500	54
	R: TATAGTCACCATTGGCTTCG		
*bla_VIM_*	F: TTTGGTCGCATATCGCAACG	501	53.5
	R: CCATTCAGCCAGATCGGCAT		
*rmpA*	F: ACTGGGCTACCTCTGCTTCA	332	56
	R: CTTGCATGAGCCATCTTTCA		
*rmpA2*	F: CTTTATGTGCAATAAG-GATGTT	451	54℃
	R: CCTCCTGGAGAGTAAGCATT		
*IroB*	F: AAGTCAAAGCAGGGGTTGCCCG	655	62℃
	R: GACGCCGACATTAAGACGCAG		
*IucA*	F: ATAAGGCGGCAATCCAAG	239	55
	R: CGCTTCACTTCTTTCACTGACAGG		
*peg-344*	F: AGAAGGCGGCAATCCAAG	694	54
	R: CGGTTCACTTCTTTCACTAGG		

### Statistical analysis

The data were analyzed using Graphpad Prism software, with categorical variables presented as frequency (n) and percentage (%). Statistical analysis was performed using SPSS 23.0 software to compare groups (e.g., biofilm vs. hypermucoviscous, hypermucoviscous vs. antibiotic resistance genes, and hypermucoviscous vs. virulence genes) that followed a normal distribution. Analyses were performed using the *χ*² test. The difference was considered statistically significant when the *P* value was less than 0.05.

## RESULTS

### Clinical sample analysis

**Fig 1:**
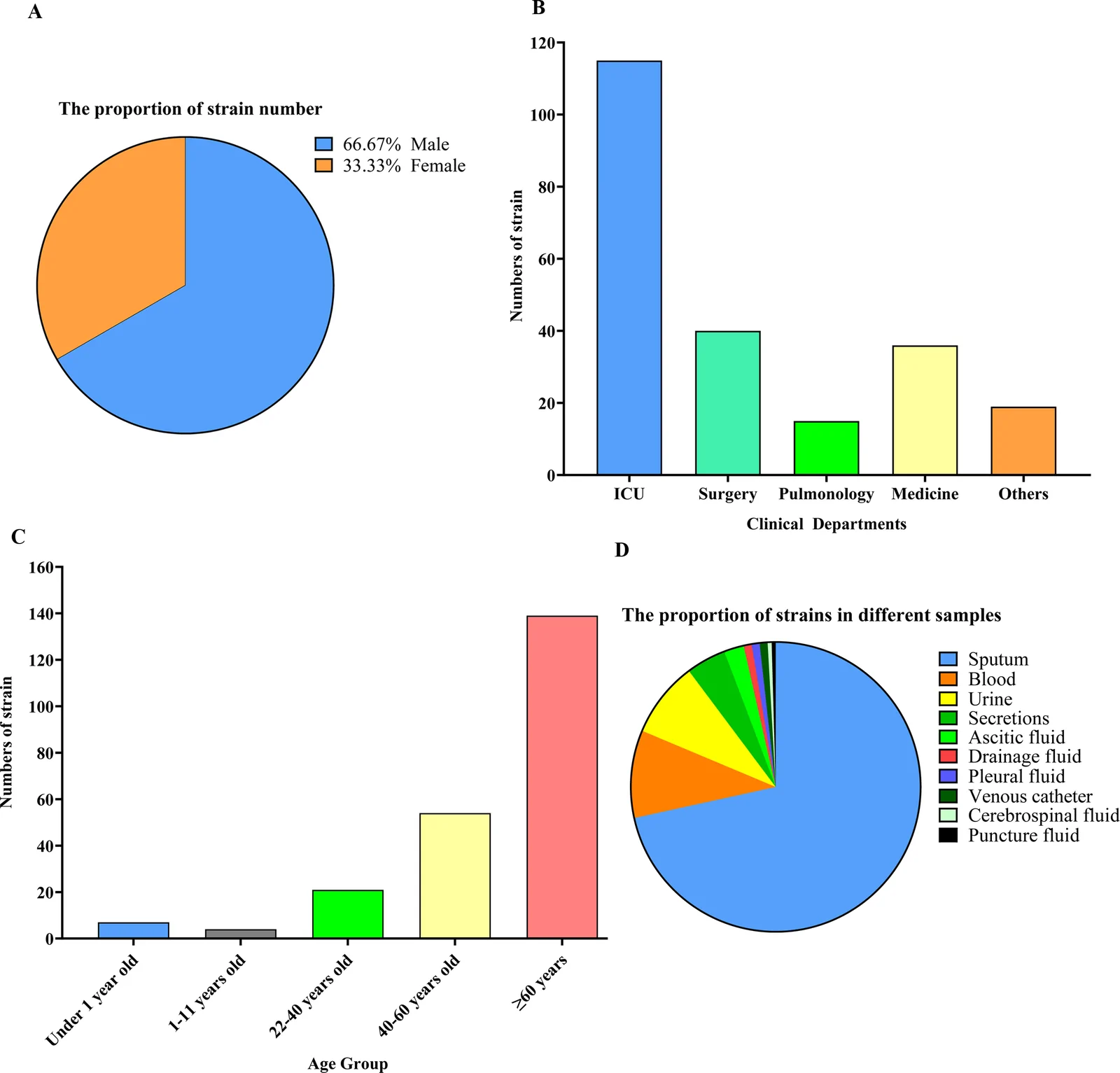
The clinical distribution of 225 strains. (**A**) Proportion of patients of different genders in the sample source. (**B**) Number of strains distributed in different clinical departments. (**C**) Age distribution of strains. (**D**) The proportion of strains in different samples.

Of the 225 clinically isolated CRKP strains, 150 strains (66.67%) were isolated from male patients (Fig. 1A). These strains were distributed across 26 departments. The intensive care unit (ICU) accounted for 51.11% (115 strains), followed by surgical departments (17.78%, 40/225) and medical departments (16.00%, 36/225) (Fig. 1B). Detailed departmental distribution is provided in Table S1. Patients aged over 60 years constituted the largest proportion (61.78%, 139/225), while pediatric patients (<12 years) accounted for only 4.89% (11/225) (Fig. 1C). Specimen source analysis indicated that sputum was the most common (71.56%, 161/225), indicating that the respiratory tract may be the primary infection site of CRKP in this region (Fig. 1D).

### Antimicrobial resistance profiles and phenotypic confirmation

All CRKP strains were resistant to Cephalosporin antibiotics (CXM, CAZ, and FOX) (100%), and Carbapenem antibiotics (IMP 100%, MEM 99.55%) (Table 1). High resistance rates (92.44%–99.56%) were noted for CRO, MEM, FEP, TZP, SCF, CIP, and LVX, while the resistance rate to TGC was only 0.88%, making it a potential salvage therapy for severe CRKP infections. Phenotypic tests confirmed that all strains were producers of ESBL; 213 strains (94.67%) were identified as producers of carbapenemase by (mCIM).

### Biofilm formation and its association with antibiotic resistance

**Fig 2:**
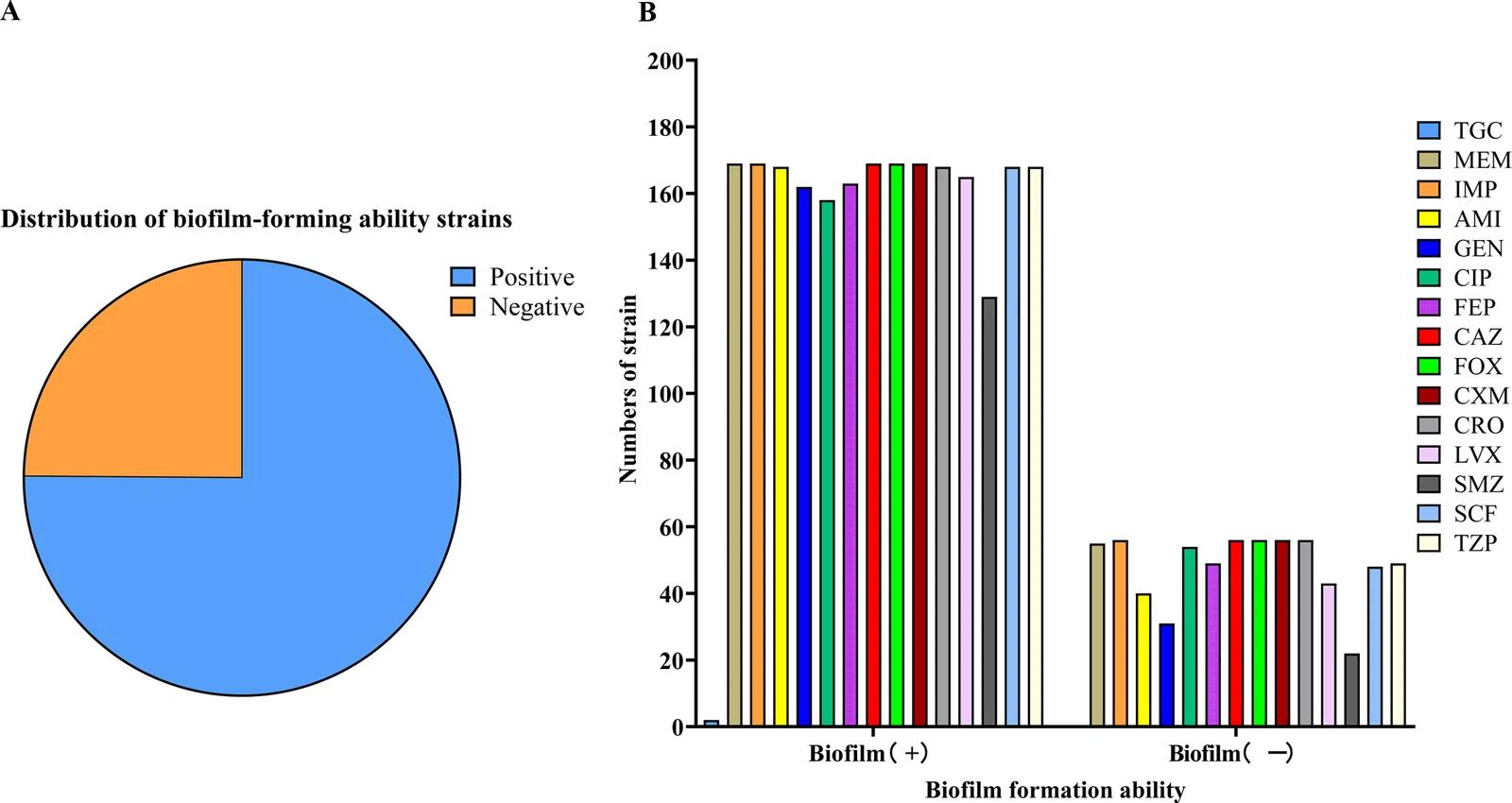
The ability of biofilm formation and its association with drug resistance. (**A**) The biofilm formation ability of the strains. (**B**) The resistant phenotype of the biofilm-positive and -negative strains.

Among the 225 CRKP strains, 169 strains (75.11%) exhibited strong biofilm-forming capacity (Fig. 2A and Table S2). Biofilm-positive strains showed higher resistance rates to AMI, GEN, LVX, and TMP-SMX (99.4%, 95.86%, 97.63%, and 76.33%) compared to biofilm-negative strains (71.43%, 55.36%, 76.39%, and 39.29%) (Fig. 2B).

### Hypermucoviscous phenotype and its correlates

**Fig 3:**
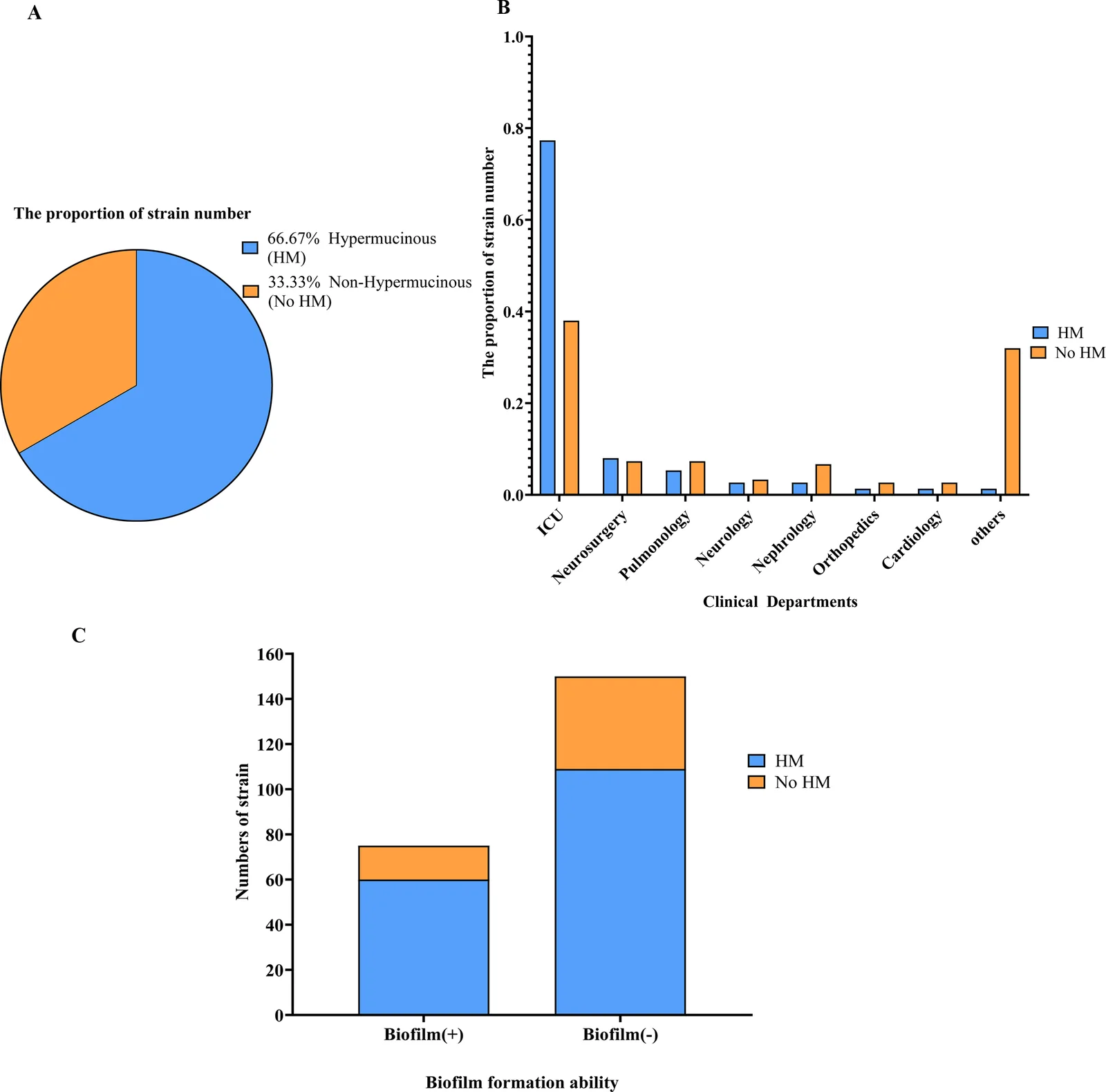
Analysis of the hypermucoviscous phenotype. (**A**) The number of HM was obtained through the string test. (**B**) The distribution of HM and non-HM in different departments. (**C**) Comparison between HM and non-HM in forming biofilms (*P* = 0.230).

In total, 75 strains (33.33%) showed positive results in the string test, which were classified as HM strains (Fig. 3A). The hypermucoviscous phenotype of *K. pneumoniae* is usually associated with virulence. Traditionally, hvKP is considered to have a hypermucoviscous phenotype and is highly pathogenic. From this perspective, 75 of the 225 CRKP strains in this study were classified as hvKP. These strains were mainly isolated from the ICU (77.33%) (Fig. 3B). Further analysis of drug resistance showed that the resistance rates of HM strains to FEP, CIP, and LVX were significantly lower than those of non-HM strains (*P* < 0.05, Table S3). No significant differences were observed in resistance to other antibiotics, including carbapenems. Additionally, the biofilm formation rate was slightly higher in HM CRKP (80%) compared to non-HM CRKP (72.67%), with no significant difference detected (*P* > 0.05, Fig. 3C).

### Distribution of antimicrobial resistance genes

**Fig 4:**
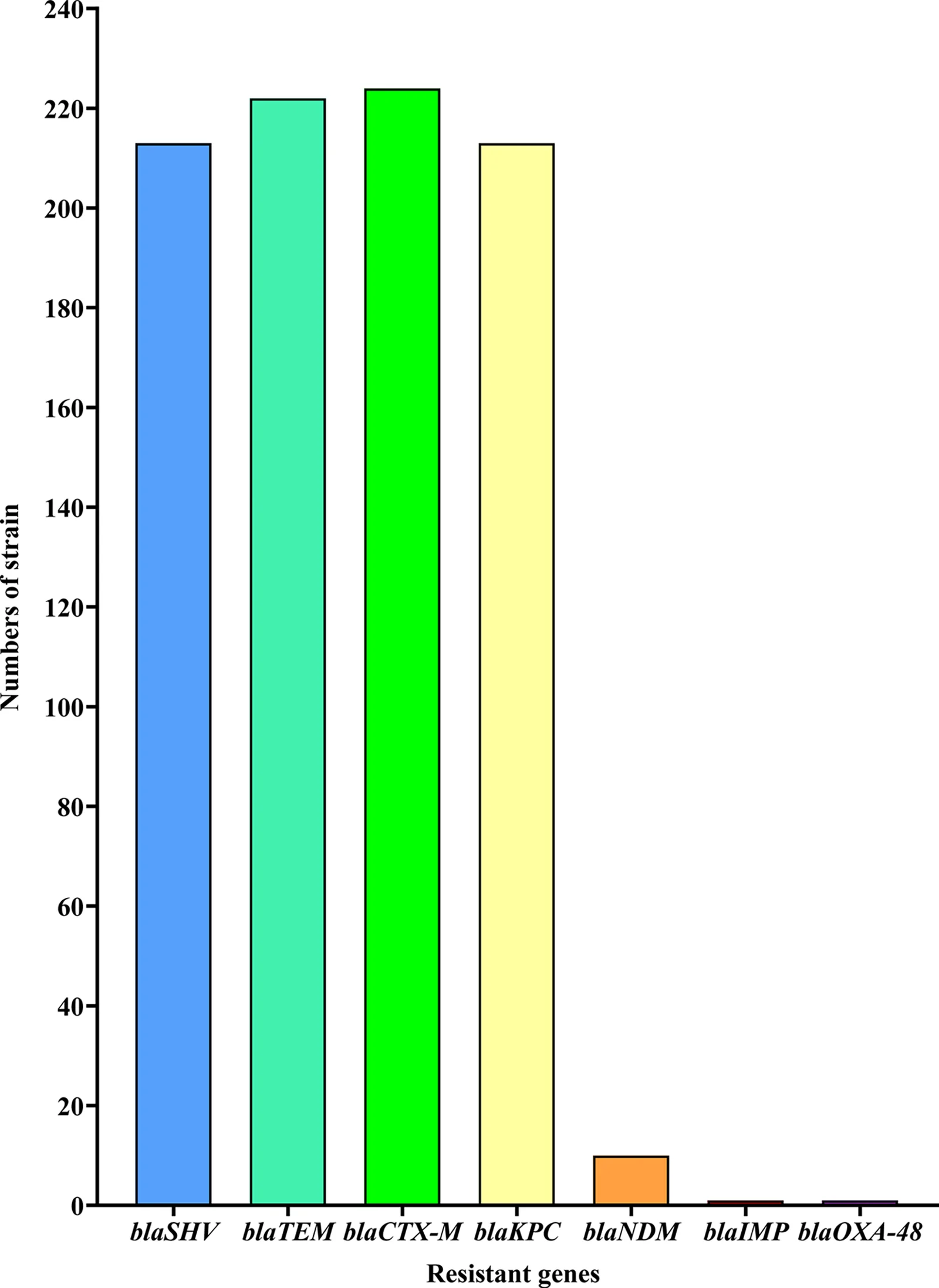
Distribution of the drug-resistant genes among the 225 CRKP strains.

The most prevalent ESBL gene was *bla_CTX-M_* (99.56%), followed by *bla_TEM_* (98.67%) and *bla_SHV_* (94.67%). Among carbapenemase genes, *bla_KPC_* was predominant (94.67%, 213/225), while *bla_NDM_* was detected in only 10 strains (4.44%) (Fig. 4).

### Virulence gene profiles and analysis of virulence-resistance associations

**Fig 5:**
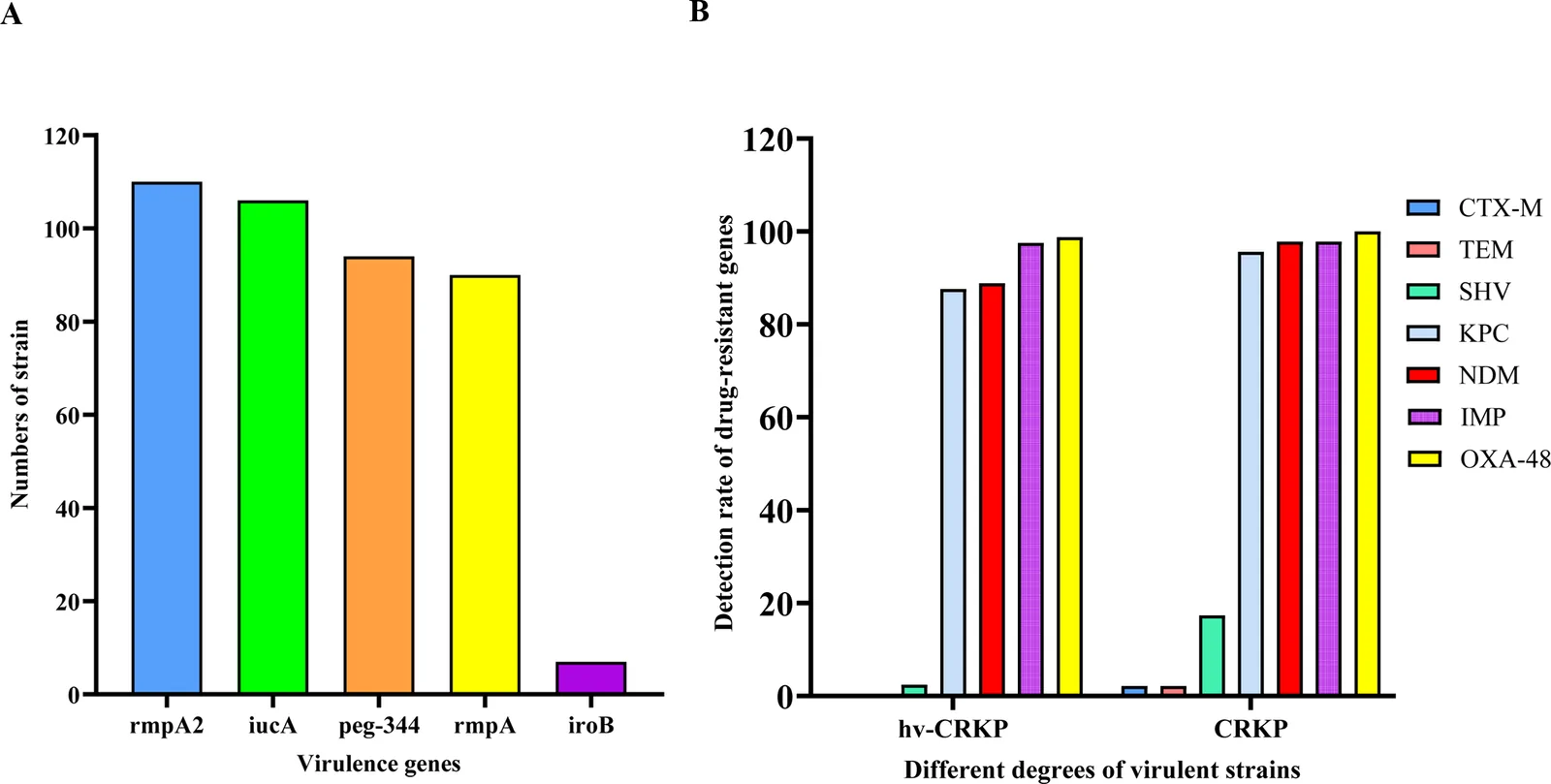
Detection of virulence-resistance genes and analysis of their relation with drug resistance. (**A**) Distribution of five main virulence genes. (**B**) Distribution of drug resistance genes in CRKP and hv-CRKP.

A subset of 127 strains (75 HM strains and 52 non-HM strains) was analyzed for virulence genes, *rmpA2* (86.61%), *iucA* (83.46%), *peg-344* (74.02%), and *rmpA* (70.87%, 90/127) as the dominant virulence markers, while *iroB* was rarely detected (5.5%) (Fig. 5A). The prevalence of *rmpA2*, *rmpA*, *peg-344*, and *iucA* was significantly higher in HM strains than in non-HM strains (*P* < 0.01, Table S4). Among them, 90 strains co-harbored at least four virulence genes and were classified as hv-CRKP at the strain level.

Comparison of the 75 HM strains with 52 non-HM strains revealed significant differences in the detection rate of certain antimicrobial resistance genes, specifically *bla_SHV_*, *bla_KPC_*, and *bla_NDM_* (*P* < 0.05) (Table S5). This indicates that the detection rates of resistance genes *bla_SHV_*, *bla_KPC_*, and *bla_NDM_* in non-HM strains were higher than those in HM strains. Additionally, compared with hv-CRKP strains, the detection rate of resistance genes in CRKP strains increased (Fig. 5B), with significantly higher detection rates of *bla_SHV_*, *bla_KPC_*, and *bla_NDM_* (*P* < 0.05, Table S6).

## DISCUSSION

CRKP infection has emerged as one of the most severe threats in clinical practice, primarily due to the limited treatment options available. This multidrug-resistant strain exhibits resistance to a wide range of antibiotics, including β-lactams and fluoroquinolones (24–26). This study presented the molecular characteristics of CRKP strains from three representative hospitals in Northern Henan Province, China—a region previously underrepresented in national surveillance networks—and elucidated the unique medical environment of these hospitals, aiming to provide a reference for clinical diagnosis and treatment.

The male predominance observed in our CRKP cohort aligns with global epidemiological patterns reported across diverse geographic regions, including North America, Europe, and Asia-Pacific healthcare systems. This gender disparity may reflect differential exposure to established risk factors, including higher rates of tobacco consumption and alcohol use among male populations, which compromise pulmonary defenses and mucociliary clearance mechanisms (27). However, we acknowledge that confounding socioeconomic and occupational variables necessitate further multivariate analysis to substantiate this association. The ICU and elderly patients (more than 60 years) were the primary reservoirs and vulnerable populations of CRKP strains, which is consistent with global reports indicating that CRKP infections primarily affect ICU patients and the elderly population(27). This may be related to impaired immunity, frequent invasive interventions, and high-intensity antimicrobial exposure among these patients (28). The predominance of respiratory specimens underscores the lung as a primary site of infection. It suggests the possibility of respiratory transmission, warranting enhanced surveillance and infection control measures in respiratory care units.

Here, CRKP exhibited near-pan resistance to multiple clinically relevant antimicrobials—ESBL-related antibiotics and carbapenems—pushing the resistance rate to or at 100%, which reflects a resistance severity exceeding national averages reported by CHINET 2024, underscoring the gravity of the therapeutic crisis. By comparison, Aminoglycosides—GEN and AMI—remained the conspicuous exception, which may be related to the absence of a β-lactam ring structure, with resistance mechanisms distinct from ESBLs or carbapenemase-mediated pathways. Additionally, their adverse effects may limit their long-term clinical use at high doses (29, 30). This may have delayed the rapid evolution of resistance to some extent. Notably, CRKP had the lowest resistance rate to TGC, which is consistent with the research on the resistance mechanisms of Gram-negative bacteria (7, 31). TGC can evade resistance mediated by *tet* (M) ribosomal protection proteins and *tet* (A-E) active efflux systems by inhibiting the binding site of the 30S ribosomal subunit (32). It suggests the potential value of TGC as a last-resort agent or for combination therapy in this region, though its clinical application must be balanced with pharmacokinetic limitations.

In this study, the vast majority of CRKP strains exhibited biofilm-forming ability, which exceeds rates reported for CRKP isolates from pediatric populations in Henan Province and surgical intensive care units in Eastern China, but approximates figures from bloodstream infection isolates in Northern China. Notably, biofilm-positive strains demonstrated significantly higher resistance to aminoglycosides (GEN, AMI) and sulfonamides (TMP-SMX). This association suggests that biofilms may confer a survival advantage in environments under selective pressure from these antibiotic classes, possibly through reduced antibiotic penetration and the presence of metabolically dormant persister cells (33). Suggesting a combined “antibiotic+antibiofilm agent” treatment strategy in clinical practice (e.g., polymyxin combined with tobramycin). Additionally, strategies for high-risk groups (e.g., ICU patients) should be implemented to reduce the risk of CRKP persistent infection mediated by biofilms.

The detection rate of HM in CRKP varies significantly across regions in China (7%–84%). This figure in our study substantially exceeds reports from Eastern China children’s hospitals, yet remains below outbreak-associated hypervirulent CRKP strains (26, 34, 35). The ICU predominance of HM strains aligns with the convergence of host susceptibility factors (prolonged hospitalization, immunosuppressive therapy, and invasive procedures) and microbial adaptation pressures (antimicrobial exposure and biofilm-promoting device surfaces) (35, 36). Additionally, the resistance rates of HM strains to FEP and fluoroquinolones (CIP and LVX) were significantly lower than those of non-HM strains, which is consistent with the conclusion reported in the literature that “resistance-virulence trade-off” (37). This may be related to the biological characteristics of HM strains, which have stronger biofilm formation ability but lower expression levels of β-lactamase. Furthermore, there was no significant difference in the biofilm formation ability of the HM strain compared to the non-HM strain, which indicates that biofilm formation and the hypermucoviscous phenotype may be independently regulated virulence mechanisms (33). This mechanistic divergence has therapeutic implications: anti-biofilm strategies may not uniformly abrogate hypermucoviscosity-associated virulence, necessitating phenotype-specific interventions.

CRKP strains can carry and spread multiple resistance determinants through mobile genetic elements such as plasmids and transposons (38). For ESBL genes, the detection rate of the *bla_CTX-M_* gene in CRKP strains was higher than that of the *bla_SHV_* and *bla_TEM_* genes, indicating that the *bla_CTX-M_* gene may be the most prevalent ESBL resistance gene in this region, which is consistent with the global trend that CTX-M-type enzymes have replaced TEM and SHV as the dominant ESBLs (39, 40). The high detection rates of *bla_SHV_* and *bla_TEM_* genes are also consistent with reports from other regions in China (41, 42). Most strains in this study co-carried *bla_SHV_* and *bla_TEM_*, reflecting the prevalence of coexistence of multidrug resistance genes. Therefore, monotherapy with β-lactam drugs should be avoided clinically. For the carbapenemase gene, detection revealed that the rates of *bla_KPC_* and *bla_NDM_* were consistent with previous studies on the predominant resistance genotypes of CRKP in the Northeast region and Henan Province (41, 43). Studies have shown that strains carrying *bla_KPC_* and *bla_NDM_* frequently accompany co-expressing virulence gene clusters (such as *rmpA* and aerobactin) (44). This “resistance-virulence” co-evolutionary pattern exacerbates the therapeutic complexity of CRKP infections.

In recent years, the number of hypervirulence strains (hv-CRKP) has increased sharply (45–47). Current research suggests that siderophore output ≥30 µg/mL, along with genes such as *iroB, iucA, peg-344*, and *rmpA/rmpA2,* is the most accurate molecular marker for defining hypervirulent *K. pneumoniae* (hvKp) (48). Aerobactin, encoded by *iucA*, capsular polysaccharides encoded by *rmpA/rmpA2*, and *peg-344* are closely associated with the hypervirulence phenotype, and CRKP strains harboring a minimum of four virulence genes were classified as hv-CRKP (20, 49, 50). The main virulence genes carried by hv-CRKP were *rmpA2*, *iucA*, *peg-344*, and *rmpA*, while *iroB* was rare. Importantly, the overall carrying rate of resistance genes in hv-CRKP was significantly lower than that in non-hv-CRKP, suggesting a significant negative correlation between hypervirulence and some resistance determinants. Similarly, HM phenotype strains had lower resistance gene-carrying rates but higher virulence gene-carrying rates than non-HM strains. These findings imply that there may be a biological trade-off or genetic incompatibility between maintaining high-level resistance gene arrays and the hypervirulence plasmidome in some strains, a phenomenon noted in recent studies (51, 52).

Although this study analyzed the association between the virulence gene profile of hv-CRKP and its phenotype, providing a theoretical foundation for understanding its pathogenic mechanisms and offering a reference for optimizing regionalized treatment strategies, several limitations should be addressed. First, the current definition of hv-CRKP does not include strains carrying 1–3 virulence genes, which may underestimate the actual epidemiological burden of hv-CRKP in this region. Additionally, virulence gene determination only covers a portion of the strains and lacks functional validation through animal experiments. Furthermore, multivariable analysis was not undertaken as the effective number of outcome events was insufficient to meet the robustness criterion of at least 10 events per variable required for reliable logistic regression (53). Future studies will expand the sample size to integrate multivariate methods and combine animal experiments with genomic analysis to refine the biological characteristic profile of hv-CRKP.

### Conclusion

This study reveals a severe resistance trend of CRKP in the northern Henan region, China, with β-lactamase genes *bla_CTX-M_* and *bla_KPC_* representing the predominant prevalent genotypes. The detection rate of hv-CRKP among the prevalent CRKP strains in this region is relatively high, and there is a significant positive correlation between hypermucoviscous phenotypes and virulence gene clusters, as well as a significant negative correlation with resistance gene burden. We attempted to construct a three-dimensional analytical framework of “antibiotic resistance gene spectrum–virulence phenotype–molecular association” to provide insights for controlling the prevalence of CRKP and clinical treatment. However, this is only a first step. Future studies will expand the sample size and combine clinical symptoms and animal experimental data in this region to further utilize transcriptomics to analyze the molecular network of resistance-virulence coevolution, providing a solid scientific basis for formulating regional hv-CRKP prevention and control guidelines.

## Data Availability

Survey data are available upon request to the corresponding author.

## SUPPLEMENTAL MATERIAL

Supplemental tables spectrum.03164-25-s0001.xlsx Tables S1 to S6.

ASM does not own the copyrights to Supplemental Material that may be linked to, or accessed through, an article. The authors have granted ASM a non-exclusive, world-wide license to publish the Supplemental Material files. Please contact the corresponding author directly for reuse.

## References

[1] Zhu J, Wang T, Chen L, Du H. 2021. Virulence factors in hypervirulent *Klebsiella pneumoniae* Front Microbiol 12:642484. doi:10.3389/fmicb.2021.642484 33897652 PMC8060575

[2] Wang M, Wang H. 2025. Carbapenem-resistant hypervirulent *Klebsiella pneumoniae*: where is it headed in the tug-of-war between virulence and resistance? eBioMedicine 114:105649. doi:10.1016/j.ebiom.2025.105649 40068322 PMC11938087

[3] Ochońska D, Klamińska-Cebula H, Dobrut A, Bulanda M, Brzychczy-Włoch M. 2021. Clonal dissemination of KPC-2, VIM-1, OXA-48-producing *Klebsiella pneumoniae* ST147 in Katowice, Poland. Pol J Microbiol 70:107–116. doi:10.33073/pjm-2021-010 33815532 PMC8008758

[4] Botelho J, Mourão J, Roberts AP, Peixe L. 2020. Comprehensive genome data analysis establishes a triple whammy of carbapenemases, ICEs and multiple clinically relevant bacteria. Microb Genom 6:mgen000424. doi:10.1099/mgen.0.000424 32841111 PMC7660259

[5] Aurilio C, Sansone P, Barbarisi M, Pota V, Giaccari LG, Coppolino F, Barbarisi A, Passavanti MB, Pace MC. 2022. Mechanisms of action of carbapenem resistance. Antibiotics 11:421. doi:10.3390/antibiotics11030421 35326884 PMC8944602

[6] Budia-Silva M, Kostyanev T, Ayala-Montaño S, Bravo-Ferrer Acosta J, Garcia-Castillo M, Cantón R, Goossens H, Rodriguez-Baño J, Grundmann H, Reuter S. 2024. International and regional spread of carbapenem-resistant *Klebsiella pneumoniae* in Europe. Nat Commun 15:5092. doi:10.1038/s41467-024-49349-z 38877000 PMC11178878

[7] Ma J, Song X, Li M, Yu Z, Cheng W, Yu Z, Zhang W, Zhang Y, Shen A, Sun H, et al.. 2023. Global spread of carbapenem-resistant *Enterobacteriaceae*: epidemiological features, resistance mechanisms, detection and therapy. Microbiol Res 266:127249. doi:10.1016/j.micres.2022.127249 36356348

[8] Ernst CM, Braxton JR, Rodriguez-Osorio CA, Zagieboylo AP, Li L, Pironti A, Manson AL, Nair AV, Benson M, Cummins K, et al.. 2020. Adaptive evolution of virulence and persistence in carbapenem-resistant *Klebsiella pneumoniae*. Nat Med 26:705–711. doi:10.1038/s41591-020-0825-4 32284589 PMC9194776

[9] Arato V, Raso MM, Gasperini G, Berlanda Scorza F, Micoli F. 2021. Prophylaxis and treatment against *Klebsiella pneumoniae*: current insights on this emerging anti-microbial resistant global threat. Int J Mol Sci 22:4042. doi:10.3390/ijms22084042 33919847 PMC8070759

[10] Simões AS, Touret T, Faria NA, Peres Ladeiro S, Costa J, Bispo S, Serrano M, Palos C, Miragaia M, Bastos Leite R, et al.. 2022. Using whole genome sequencing to investigate a mock-outbreak of carbapenem-resistant *Klebsiella pneumoniae* in real-time. Acta Med Port 35:36–41. doi:10.20344/amp.15174 34755594

[11] Hou B, Niu X, Yu Q, Wang W. 2025. Epidemiological trends and drug resistance patterns of carbapenem-resistant gram-negative bacteria: a retrospective study in a tertiary hospital in China (2019-2024). Infect Drug Resist 18:2867–2880. doi:10.2147/IDR.S518461 40492239 PMC12147788

[12] Muresu N, Deiana G, Dettori M, Palmieri A, Masia MD, Cossu A, D’Avino C, Sechi I, Del Rio A, Piana A, et al.. 2023. Infection prevention control strategies of New Delhi metallo-β-lactamase producing *Klebsiella pneumoniae*. Healthcare (Basel) 11:2592. doi:10.3390/healthcare11182592 37761789 PMC10530878

[13] Surgers L, Boyd A, Girard P-M, Arlet G, Decré D. 2019. Biofilm formation by ESBL-producing strains of *Escherichia coli* and *Klebsiella pneumoniae*. Int J Med Microbiol 309:13–18. doi:10.1016/j.ijmm.2018.10.008 30385204

[14] Hu F, Pan Y, Li H, Han R, Liu X, Ma R, Wu Y, Lun H, Qin X, Li J, et al.. 2024. Carbapenem-resistant *Klebsiella pneumoniae* capsular types, antibiotic resistance and virulence factors in China: a longitudinal, multi-centre study. Nat Microbiol 9:814–829. doi:10.1038/s41564-024-01612-1 38424289 PMC10914598

[15] Heng ST, Chen SL, Wong JGX, Lye DC, Ng TM. 2018. No association between resistance mutations, empiric antibiotic, and mortality in ceftriaxone-resistant *Escherichia coli* and *Klebsiella pneumoniae* bacteremia. Sci Rep 8:12785. doi:10.1038/s41598-018-31081-6 30143706 PMC6109088

[16] Humphries R, Bobenchik AM, Hindler JA, Schuetz AN, McAdam AJ. 2021. Overview of changes to the clinical and laboratory standards institute performance standards for antimicrobial susceptibility testing*,* M100, 31st edition. J Clin Microbiol 59:e0021321. doi:10.1128/JCM.00213-21 34550809 PMC8601225

[17] Hu Q, Zhou Y, Zhu Y, Ma Z, Li T, Yao S, Pan J, Shi M, Su F, Shen B, et al.. 2025. High-throughput clinical antimicrobial susceptibility testing and drug-resistant subpopulation detection in gram-negative bacteria. Microbiol Spectr 13:e0001125. doi:10.1128/spectrum.00011-25 40470969 PMC12211026

[18] Kaderabkova N, Mahmood AJS, Mavridou DAI. 2024. Antibiotic susceptibility testing using minimum inhibitory concentration (MIC) assays. NPJ Antimicrob Resist 2:37. doi:10.1038/s44259-024-00051-6 39843555 PMC11721449

[19] Stepanovic S, Vukovic D, Hola V, Di Bonaventura G, Djukic S, Cirkovic I, Ruzicka F. 2007. Quantification of biofilm in microtiter plates: overview of testing conditions and practical recommendations for assessment of biofilm production by Staphylococci. APMIS 115:891–899. doi:10.1111/j.1600-0463.2007.apm_630.x 17696944

[20] Wang X, Wang J, Jiang X, Huang Z, Huang L, Wei Q, Zhang L. 2025. Molecular epidemiological analysis and research on resistance and virulence of carbapenem-resistant *Klebsiella pneumoniae* in a tertiary hospital from 2016 to 2023. BMC Microbiol 25:217. doi:10.1186/s12866-025-03888-7 40234763 PMC12001477

[21] Russo TA, MacDonald U, Papasian CJ. 2020. The *Galleria mellonella* infection model does not accurately differentiate between hypervirulent and classical *Klebsiella pneumoniae*. mSphere 5. doi:10.1128/mSphere.00850-19 PMC6952204 31915230

[22] Endimiani A, Depasquale JM, Forero S, Perez F, Hujer AM, Roberts-Pollack D, Fiorella PD, Pickens N, Kitchel B, Casiano-Colon AE, et al.. 2009. Emergence of blaKPC-containing *Klebsiella pneumoniae* in a long-term acute care hospital: a new challenge to our healthcare system. J Antimicrob Chemother 64:1102–1110. doi:10.1093/jac/dkp327 19740911 PMC2760463

[23] Illenseher MS, Hentschker C, Gesell Salazar M, Busch LM, Zierke L, Reder A, Michalik S, Völker U, Hammerschmidt S, Surmann K. 2025. Global quantitative proteome analysis of a multi-resistant *Klebsiella pneumoniae* strain. Front Microbiol 16:1528869. doi:10.3389/fmicb.2025.1528869 40458706 PMC12127431

[24] Gu D, Dong N, Zheng Z, Lin D, Huang M, Wang L, Chan E-C, Shu L, Yu J, Zhang R, et al.. 2018. A fatal outbreak of ST11 carbapenem-resistant hypervirulent *Klebsiella pneumoniae* in a chinese hospital: a molecular epidemiological study. Lancet Infect Dis 18:37–46. doi:10.1016/S1473-3099(17)30489-9 28864030

[25] Yang X, Sun Q, Li J, Jiang Y, Li Y, Lin J, Chen K, Chan EW-C, Zhang R, Chen S. 2022. Molecular epidemiology of carbapenem-resistant hypervirulent *Klebsiella pneumoniae* in China. Emerg Microbes Infect 11:841–849. doi:10.1080/22221751.2022.2049458 35236251 PMC8942559

[26] Li Y, Li D, Xue J, Ji X, Shao X, Yan J. 2021. The epidemiology, virulence and antimicrobial resistance of invasive *Klebsiella pneumoniae* at a children’s medical center in eastern China. IDR Volume 14:3737–3752. doi:10.2147/IDR.S323353 PMC8449645 34548798

[27] Chen TA, Chuang YT, Lin CH. 2024. A decade-long review of the virulence, resistance, and epidemiological risks of *Klebsiella pneumoniae* in ICUs. Microorganisms 12:2548. doi:10.3390/microorganisms12122548 39770751 PMC11678397

[28] Martin RM, Bachman MA. 2018. Colonization, infection, and the accessory genome of *Klebsiella pneumoniae* Front Cell Infect Microbiol 8:4. doi:10.3389/fcimb.2018.00004 29404282 PMC5786545

[29] Serrano-Arias B, Araya-Zúñiga A, Waterhouse-Garbanzo J, Rojas-Barrantes Z, Arguedas-Chacón S, Zavaleta-Monestel E. 2024. A comprehensive review of sulfonamide hypersensitivity: implications for clinical practice. Clinic Rev Allerg Immunol 65:433–442. doi:10.1007/s12016-023-08978-w 38175321

[30] Beig M, Aghamohammad S, Majidzadeh N, Asforooshani MK, Rezaie N, Abed S, Khiavi EHG, Sholeh M. 2024. Antibiotic resistance rates in hypervirulent *Klebsiella pneumoniae* strains: a systematic review and meta-analysis. J Glob Antimicrob Resist 38:376–388. doi:10.1016/j.jgar.2024.06.018 39069234

[31] Li Q, Zhou X, Yang R, Shen X, Li G, Zhang C, Li P, Li S, Xie J, Yang Y. 2024. Carbapenem-resistant gram-negative bacteria (CR-GNB) in ICUs: resistance genes, therapeutics, and prevention – a comprehensive review. Front Public Health 12. doi:10.3389/fpubh.2024.1376513 PMC11004409 38601497

[32] Yaghoubi S, Zekiy AO, Krutova M, Gholami M, Kouhsari E, Sholeh M, Ghafouri Z, Maleki F. 2022. Tigecycline antibacterial activity, clinical effectiveness, and mechanisms and epidemiology of resistance: narrative review. Eur J Clin Microbiol Infect Dis 41:1003–1022. doi:10.1007/s10096-020-04121-1 33403565 PMC7785128

[33] Li L, Gao X, Li M, Liu Y, Ma J, Wang X, Yu Z, Cheng W, Zhang W, Sun H, et al.. 2024. Relationship between biofilm formation and antibiotic resistance of *Klebsiella pneumoniae* and updates on antibiofilm therapeutic strategies. Front Cell Infect Microbiol 14. doi:10.3389/fcimb.2024.1324895 PMC10920351 38465230

[34] Wang H, Jia Z, Li X, Hao Y, Zhang J, Zhao X, Li H, Jin S. 2025. Molecular characteristics and phenotypical analysis of carbapenem-resistant *K. pneumoniae* in the Lüliang region, Shanxi province. Infect Drug Resist 18:2911–2921. doi:10.2147/IDR.S512203 40502539 PMC12153953

[35] Ma J, Gao K, Li M, Zhou J, Song X, Zhang Y, Yu Z, Yu Z, Cheng W, Zhang W, et al.. 2024. Epidemiological and molecular characteristics of carbapenem-resistant *Klebsiella pneumoniae* from pediatric patients in Henan, China. Ann Clin Microbiol Antimicrob 23:98. doi:10.1186/s12941-024-00757-5 39511610 PMC11545200

[36] Tian C, Shi Y, Ren L, Huang D, Wang S, Zhao Y, Fu L, Bai Y, Xia D, Fan X. 2023. Emergence of IS26-mediated pLVPK-like virulence and NDM-1 conjugative fusion plasmid in hypervirulent carbapenem-resistant *Klebsiella pneumoniae*. Infect Genet Evol 113:105471. doi:10.1016/j.meegid.2023.105471 37353184

[37] Xu Q, Sun R, Liu X, Heng H, Yang X, Xie M, Yang C, Ye L, Chan E-C, Zhang R, et al.. 2025. Global dissemination of conjugative virulence plasmids co-harboring hypervirulence and multidrug resistance genes in *Klebsiella pneumoniae*. mSystems 10. doi:10.1128/msystems.01675-24 PMC12013265 40130870

[38] Mbelle NM, Feldman C, Sekyere JO, Maningi NE, Modipane L, Essack SY. 2020. Pathogenomics and evolutionary epidemiology of multi-drug resistant clinical *Klebsiella pneumoniae* isolated from Pretoria, South Africa. Sci Rep 10:1232. doi:10.1038/s41598-020-58012-8 31988374 PMC6985128

[39] Quan J, Zhao D, Liu L, Chen Y, Zhou J, Jiang Y, Du X, Zhou Z, Akova M, Yu Y. 2017. High prevalence of ESBL-producing *Escherichia coli* and *Klebsiella pneumoniae* in community-onset bloodstream infections in China. J Antimicrob Chemother 72:273–280. doi:10.1093/jac/dkw372 27624571

[40] Zhang J, Zhou K, Zheng B, Zhao L, Shen P, Ji J, Wei Z, Li L, Zhou J, Xiao Y. 2016. High prevalence of ESBL-producing *Klebsiella pneumoniae* causing community-onset infections in China. Front Microbiol 7. doi:10.3389/fmicb.2016.01830 PMC5109008 27895637

[41] Zhao H, He Z, Li Y, Sun B. 2022. Epidemiology of carbapenem-resistant *Klebsiella pneumoniae* ST15 of producing KPC-2, SHV-106 and CTX-M-15 in Anhui, China. BMC Microbiol 22. doi:10.1186/s12866-022-02672-1 PMC9624029 36319965

[42] Castanheira M, Simner PJ, Bradford PA. 2021. Extended-spectrum β-lactamases: an update on their characteristics, epidemiology and detection. JAC Antimicrob Resist 3:dlab092. doi:10.1093/jacamr/dlab092 34286272 PMC8284625

[43] Wang Q, Wang X, Wang J, Ouyang P, Jin C, Wang R, Zhang Y, Jin L, Chen H, Wang Z, et al.. 2018. Phenotypic and genotypic characterization of carbapenem-resistant *Enterobacteriaceae*: data from a longitudinal large-scale CRE study in China (2012-2016). Clin Infect Dis 67:S196–S205. doi:10.1093/cid/ciy660 30423057

[44] Liu X, Zhang J, Li Y, Shen Q, Jiang W, Zhao K, He Y, Dai P, Nie Z, Xu X, et al.. 2019. Diversity and frequency of resistance and virulence genes in bla_KPC_ and bla_NDM_ co-producing *Klebsiella pneumoniae* strains from China. Infect Drug Resist Volume 12:2819–2826. doi:10.2147/IDR.S214960 PMC6750849 31571938

[45] Ballén V, Gabasa Y, Ratia C, Ortega R, Tejero M, Soto S. 2021. Antibiotic resistance and virulence profiles of *Klebsiella pneumoniae* strains isolated from different clinical sources. Front Cell Infect Microbiol 11:738223. doi:10.3389/fcimb.2021.738223 34540722 PMC8440954

[46] Kocsis B. 2023. Hypervirulent *Klebsiella pneumoniae*: an update on epidemiology, detection and antibiotic resistance. Acta Microbiol Immunol Hung 70:278–287. doi:10.1556/030.2023.02186 38047929

[47] Bhide PP, Ketkar AA, Almeligy A, Ricca A. 2022. *Klebsiella* invasive syndrome: a challenging diagnosis. BMJ Case Rep 15:e251977. doi:10.1136/bcr-2022-251977 PMC9660613 36368733

[48] Tang Y, Du P, Du C, Yang P, Shen N, Russo TA, Liu C. 2025. Genomically defined hypervirulent *Klebsiella pneumoniae* contributed to early-onset increased mortality. Nat Commun 16. doi:10.1038/s41467-025-57379-4 PMC11873152 40025046

[49] Clegg S, Murphy CN, Mulvey MA, Stapleton AE, Klumpp DJ. 2016. Epidemiology and virulence of *Klebsiella pneumoniae*. Microbiol Spectr 4. doi:10.1128/microbiolspec.UTI-0005-2012 26999397

[50] Russo TA, Olson R, Fang C-T, Stoesser N, Miller M, MacDonald U, Hutson A, Barker JH, La Hoz RM, Johnson JR. 2018. Identification of biomarkers for differentiation of hypervirulent *Klebsiella pneumoniae* from classical *K. pneumoniae*. J Clin Microbiol 56:e00776-18. doi:10.1128/JCM.00776-18 29925642 PMC6113484

[51] Chen KD, Chen W, Zhang Q, Li Q. 2024. The impact of antibiotic induction on virulence and antibiotic resistance in *Klebsiella pneumoniae*: a comparative study of CSKP and CRKP strains. Front Microbiol 15:1498779. doi:10.3389/fmicb.2024.1498779 39498139 PMC11532078

[52] Choby JE, Howard-Anderson J, Weiss DS. 2020. Hypervirulent *Klebsiella pneumoniae* - clinical and molecular perspectives. J Intern Med 287:283–300. doi:10.1111/joim.13007 31677303 PMC7057273

[53] Peduzzi P, Concato J, Kemper E, Holford TR, Feinstein AR. 1996. A simulation study of the number of events per variable in logistic regression analysis. J Clin Epidemiol 49:1373–1379. doi:10.1016/s0895-4356(96)00236-3 8970487

